# Male Investments in High Quality Sperm Improve Fertilization Success, but May Have Negative Impact on Offspring Fitness in Whitefish

**DOI:** 10.1371/journal.pone.0137005

**Published:** 2015-09-21

**Authors:** Jukka Kekäläinen, Carles Soler, Sami Veentaus, Hannu Huuskonen

**Affiliations:** 1 Department of Biology, University of Eastern Finland, Joensuu, Finland; 2 Centre for Evolutionary Biology, School of Animal Biology, University of Western Australia, Crawley, Australia; 3 Departament de Biologia Funcional i Antropologia Física, Universitat de València, Burjassot, Spain; Universidad Nacional Autónoma de México, MEXICO

## Abstract

Many ejaculate traits show remarkable variation in relation to male social status. Males in disfavoured (subordinate) mating positions often invest heavily on sperm motility but may have less available resources on traits (e.g., secondary sexual ornaments) that improve the probability of gaining matings. Although higher investments in sperm motility can increase the relative fertilization success of subordinate males, it is unclear whether status-dependent differences in sperm traits could have any consequences for offspring fitness. We tested this possibility in whitefish (*Coregonus lavaretus* L.) by experimentally fertilizing the eggs of 24 females with the sperm of either highly-ornamented (large breeding tubercles, dominant) or less-ornamented (small tubercles, subordinate) males (split-clutch breeding design). In comparison to highly-ornamented individuals, less-ornamented males had higher sperm motility, which fertilized the eggs more efficiently, but produced embryos with impaired hatching success. Also offspring size and body condition were lower among less-ornamented males. Furthermore, sperm motility was positively associated with the fertilization success and offspring size, but only in highly-ornamented males. Together our results indicate that male investments on highly motile (fertile) sperm is not necessarily advantageous during later offspring ontogeny and that male status-dependent differences in sperm phenotype may have important effects on offspring fitness in different life-history stages.

## Introduction

Sperm competition theory predicts that reproductive males face a trade-off between number of matings gained and resources allocated on a single mating event or an ejaculate [1,2]. Accordingly, male investments on traits improving the probability of gaining matings (e.g. secondary sexual ornaments) are expected to lead to a reduction in sperm quality or quantity [3,4,5,6,7]. Furthermore, several studies have demonstrated that males in disfavoured mating roles (e.g. subordinates) invest more on their semen than males in more favoured mating roles [8,9,10,11,12,13]. Thus, males have been shown to adjust their sperm characteristics according to their social status [14,15,16,17,18,19]. As fertilization success under sperm competition is largely dependent on ejaculate quantity and/or quality (e.g. [20,21,22]), sperm plasticity may allow subordinate individuals to compensate their reproductive disadvantage in relation to dominant individuals (e.g. [13,23,24]).

Young et al. [24] recently tested this possibility in Chinook salmon (*Oncorhynchus tshawytscha*). After controlling for the differences in sperm quantity and distance of sperm release from female gametes, authors showed that in sperm competition situation subordinate (sneaker) males fertilized ca. 1.35 times more eggs than dominant males. Vladić et al. [13] conducted similar experiment in precociously mature parr (subordinates) and anadromous (dominant) males in Atlantic salmon (*Salmo salar*) and found even larger difference: fertilization success of subordinate individuals was 3.6 times higher than in dominant males. The authors also found that fertilization success in single fertilizations was strongly positively correlated with fertilization success of the same eggs under sperm competition. Similar association has been demonstrated also in other externally fertilizing species, the Moor frog (*Rana arvalis*) [25]. Thus, largely similar selection forces seem to determine male fertilization success both in single fertilizations and when ejaculates are competing against each other.

Several studies have demonstrated that improved fertilization success of subordinate individuals is often associated with higher sperm motility (e.g. [7,10,14,15,16,17,19]). Supporting this view sperm motility has been shown to be one of the most important factors predicting the paternity of the males, especially in externally fertilizing fish, such as salmonids [26,27,28]. “Good sperm” hypothesis predicts that sperm that are effective in fertilizing the eggs are also more effective in producing viable offspring. In other words, male sperm competition ability should correlate with the fitness of his offspring [29]. Thus, one of the key assumptions of the hypothesis is the direct genetic correlation between sperm quality and offspring viability. If such an association really does exist we could theoretically predict that sperm of the subordinate males is superior to dominant males not only because sperm of subordinate males fertilize the eggs more efficiently, but also because sperm of subordinate males will produce superior offspring. However, selection environments often vary considerably between different life-history stages [30] and selection acting on the earlier life-history trait (e.g. sperm fertilization efficiency) does not necessarily predict offspring fitness in the later stages of offspring ontogeny (e.g. [31]). Furthermore, it has recently been argued that environmental (and thus also status-dependent) plasticity in sperm traits may have previously unknown consequences for offspring fitness (non-genetic paternal effects: [32,33,34,35], which may further modify the genetic association between sperm quality and offspring fitness [34].

Status- and mating tactic- dependent differences in sperm traits have been well demonstrated in externally fertilizing fish such as salmonids (e.g. [10,15,24]). Earlier studies have also shown that male mating tactic (territorial vs. precociously mature males) can have important consequences for offspring fitness (e.g. [36,37,38]). However, the post-fertilization fitness consequences of male status and associated status-dependent differences in sperm traits have remained poorly studied [35]. Whitefish (*Coregonus lavaretus* (L.)) females produce large number of eggs that are externally fertilized. During the breeding season males (and in many populations also females) develop secondary sexual traits, keratinized, pale-coloured epidermal breeding tubercles [39,40,41]. Previous studies have demonstrated that the size and/or number of the whitefish male breeding tubercles are positively linked to male dominance status [42]), embryo survival [40], offspring first-feeding success [43] as well as offspring swimming performance and predator avoidance ability [44]. Furthermore, sperm swimming velocity is lower in dominant (larger) males [17,42], which indicates that both large tubercles and high dominance status are associated with slower sperm swimming velocity.

The primary aim of the present study was to clarify the fitness consequences of status-dependent differences in sperm quality (motility) in whitefish. We used split-clutch (maternal half sibling) breeding design and fertilized the eggs of 24 females with similar quantity of sperm of either less-ornamented (no breeding tubercles/small tubercles, subordinate) or highly-ornamented (large tubercles, dominant) males. Eggs were incubated until hatching and then hatched larvae were reared for ca. 20 weeks in standardized laboratory conditions. This breeding design allowed us to control potentially confounding maternal and paternal material benefits and clarify the effect of sperm motility variation on fertilization success and embryo mortality as well as on offspring post-hatching fitness (body size and condition).

## Material and Methods

### Fish collection and formation of male groups

*Coregonus* sp. aggregate for their spawning areas once a year and typically spawn in large aggregations, suggesting that sperm competition is intense ([42], personal observations by the authors). In the present population spawning season typically lasts ca. 3–4 weeks, from late October to middle November. Parental fish of *Coregonus lavaretus* were gill-netted (mesh sizes: 25–40 mm) from their natural spawning sites in Lake Pyhäselkä, Eastern Finland (62°33'N 29°43'E) on 29^th^ and 30^th^ October 2012. In order to minimize the stress of captured individuals, all gill-nets were checked twice daily. Experimental gill-netting was conducted according the permit issued by the Northern Carelia Employment and Economic Development Centre, Finland (permit no. 27/5716/06). Immediately after capturing, the fish were carefully released and placed into 80 l plastic tubs filled with well-oxygenated lake water and transported alive into nearby University of Eastern Finland, Joensuu campus, where all the experiments were conducted. In the laboratory, fish were euthanized with an overdose of tricaine methanesulfonate (Finquel^®^, Argent Chemical Laboratories, Redmond, WA, USA). All the individuals were measured for total length (L_T_) and fresh mass (M_T_) and their condition was determined using an equation: *K = 100* * *M*_*T*_ * *L*_*T*_^*-b*^, where *b* was obtained as a regression slope of *lnM*_*T*_ and *lnL*_*T*_ [45]. In addition, age of parental fish was later determined by counting the annual growth rings of the scales using a microfilm reader. Prior to fertilizations two independent observers ranked the breeding ornamentation (tubercle size) of the males on the scale 0–3 (0 = no tubercles, 1 = small tubercles; 2 = medium-sized tubercles, 3 = large tubercles). Then based on their breeding ornamentation we formed a total of 24 male pairs, which was estimated to provide sufficient sample size for the detection of potential between-group differences in testable parameters. Within a male pair, one male represented highly-ornamented individual (mean rank 2.77, S.D. 0.38), whereas the other male was either lacking tubercles completely or had only small tubercles (mean rank 0.92, S.D. 0.36; less-ornamented individual). Within each pair, males were size-matched by length. Accordingly, the mean total length and condition of the two male groups did not differ (linear mixed model, length: d.f. = 23, t = 1.499, P = 0.147; condition: d.f. = 46, t = 1.801, P = 0.078). Highly-ornamented males had larger fresh mass than less-ornamented individuals (d.f = 23, t = 2.092, P = 0.048). The mean age of the males (determined from scales) was almost identical in the two groups: 4.7 (± 0.70 S.D.) and 4.8 (± 0.88 S.D.) years, in highly-ornamented and less-ornamented males, respectively. The females were on average 4.9 (± 0.85 S.D.) years old.

### Artificial fertilization and maintenance of the eggs

The milt from both males within each 24 pairs was simultaneously stripped into cooled Petri dishes and then used to fertilize the eggs of the same haphazardly selected female. Each female was used only once (i.e. for one male pair), resulting in 24 fertilization blocks in total. Prior to fertilizations, male spermatocrit (sperm volume) was measured by centrifuging sperm samples for 10 minutes (11 000 rpm) in micro-hematocrit centrifuge (SpinCrit, http://www.spincritcentrifuge.com). By using highest male-specific spermatocrit as a reference value, we equalized number of sperm cells across the 48 fertilizations. The final sperm volume in all fertilizations was 3 μl of pure spermatozoa (or equivalent to 10 μl of milt with 30% spermatocrit). Fertilizations were made on Petri dishes, by injecting the sperm with micropipette directly on the freshly stripped eggs (ca. 200 eggs/male). Immediately after this, 50 ml of Lake Pyhäselkä water was poured on the Petri dish and each dish was gently shaken for 5 s to allow the eggs to be fertilized. Between 5–8^th^ November 2012, the fertilization rate of the eggs of each male-female combination was determined (by identifying visible cell division) from a random sample of 50 eggs using microscopic examination. Then the remaining eggs were randomly divided in three individual incubating containers placed in three 600-l water tanks filled with 4°C aerated and non-chlorinated tap water. Eggs were incubated in these containers until hatching in April 2013. Mortality of the eggs was followed weekly by counting and removing the dead eggs. In addition to total egg mortality (egg mortality from fertilization to hatching), also egg hatching mortality (mortality during the hatching period, 1^st^ March to 2^nd^ April 2013) was determined. Hatched larvae were euthanized with an overdose of tricaine methanesulfonate and were then preserved in a solution of 70% ethanol and 1% neutralized formalin for later length measurements (larvae total length at hatching).

### Creation and maintenance of the juvenile groups

On 21^st^ March a haphazard sample of six hatched larvae from each of the 48 male-female combination from each of the three replicate tanks were pooled into two groups (6 larvae × 48 × 3 = 864 larvae in total and 432 larvae/male group). Individuals in both of the above-mentioned groups were further divided into two replicate 45-l rectangular flow-through glass aquaria, resulting 216 fish/aquarium. Offspring were raised in these aquaria at 16°C until 5^th^ August (ca. 20 weeks) when fish were approximately 65 mm in length. The fish were fed ad libitum on *Artemia* nauplii once a day (21^st^ March to 6^th^ May 2013) and then (7^th^ May to 5^th^ August) on commercial dry food (Biomar Inicio plus, Biomar, Brande, Denmark). The aquaria were cleaned five times/week by siphoning faeces and uneaten food particles from the bottom. Mortality of the offspring (juvenile mortality) was recorded during cleaning operations. After the experiment, all fish were euthanized with an overdose of tricaine methanesulfonate and their total length, body mass and condition factor (see above) were determined.

### Sperm measurements

Male sperm motility was measured using Computer Assisted Sperm Analysis, CASA (Integrated Semen Analysis System, ISAS v1: Proiser, Valencia, Spain) with B/W CCD camera (capture rate 60 frames/s) and negative phase contrast microscope (100 × magnification). Sperm motility analyses were performed by adding 0.1 μl of sperm dilution to Leja 2-chamber (chamber height 20 μm, volume 6 μl) microscope slides (Leja, Nieuw-Vennep, The Netherlands) and by activating sperm with 3 μl of 4°C Lake Pyhäselkä water. Sperm motility parameters were measured 10 s after activation (three replicate measurements/male). Measured sperm motility parameters were (1) straight line velocity (VSL); (2) curvilinear velocity (VCL); (3) average path velocity (VAP); (4) straightness of the swimming trajectory (STR); (5) linearity of the swimming trajectory (LIN); and (6) the percentage of static (immobile); and (7) rapid sperm cells. All experiments and procedures have been approved by the Finnish National Animal Experiment Board (license number: ESAVI/1906/04.10.03/2012). All sections of this report adhere to the ARRIVE Guidelines for reporting animal research [46]. A completed ARRIVE guidelines checklist is included in S1 Checklist.

### Statistical analyses

To reduce the number of correlated variables, we conducted principal component analyses (PCA) for above-mentioned seven sperm parameters (Table 1). PCA produced two principal components (with eigenvalue > 1). The first component (PC1, all variable loadings > 0.8) described sperm motility: Swimming velocity (VSL, VCL and VAP; measurements 1–3) and proportion (%) of static and rapid cells (measurements 6–7), whereas the second component (PC2, both variable loadings > 0.9) was associated with the curvature (form) of the swimming trajectory (STR and LIN; measurements 4–5). The effect of male group (highly-ornamented or less-ornamented males, within-subject factor) on egg fertilization success, total egg mortality and size of the newly-hatched larvae was studied by fitting linear mixed models (LMM, with REML method) where fertilization success, total egg mortality or larvae total length was acted as a response variable, male group as a fixed effect and fertilization block (1 block = 1 female and 2 males) as a random effect. The effect of male group on egg hatching mortality was tested using a generalized linear mixed model (GLMM), with negative binomial error distribution, log link function and by adding male group as a within-subject factor (fixed effect) and fertilization block (1 female and 2 males) as a random effect in the model. GLMM was used since the response variable was not normally distributed and could not be normalized with transformations. Final selection of error distribution was based on lowest Akaike’s information criteria (AIC) value between three candidate distributions (negative binomial, quasi-poisson and poisson). The difference between male groups in sperm traits (PC1: motility and PC2: swimming trajectory) was studied as above, but now using these two PC-scores as response variables in LMM.

**Table 1:** Results of Principal Component Analysis (PCA) for measured sperm traits.

Measurement	PC1	PC2
VCL	**0.980**	0.122
VSL	**0.830**	0.549
VAP	**0.967**	0.227
% of rapid sperm	**0.957**	0.079
% of static sperm	**-0.899**	-0.096
STR	0.029	**0.995**
LIN	0.267	**0.959**
Eigenvalue	4.96	1.72
% of variance	70.8	24.5
Total variance (%)		95.3

First component (PC1) describes sperm motility and second component (PC2) curvature of sperm swimming tracks.

The association between sperm motility and egg mortality was studied in LMM (with REML), where sperm motility (PC1) acted as a response variable, male group, fertilization success, hatching mortality and total egg mortality as fixed effects and fertilization block as a random effect. Initial model was simplified first by removing all the non-significant interactions between fixed effects (P > 0.05, in all cases) and then by using AIC. The final model (based on lowest AIC) included male group, fertilization success and fertilization block. The association between sperm motility and larvae total length was studied similarly, but using larvae total length as a fixed effect. Finally, juvenile mortality between male groups was tested using Fisher’s Exact test and the size and condition differences with One-Way ANOVAs. Model fits were verified graphically using Q-Q plots and residual plots. Mixed model (LMM and GLMM) analyses were conducted using lmerTest (version 2.0–20) and glmmADMB (version 0.8.0) packages (respectively) in R (version 3.1.2, [47]). All other analyses were performed using SPSS (version 21.0).

## Results

### Sperm motility, fertilization success, egg mortality and larval size differences between male groups

**Fig 1:**
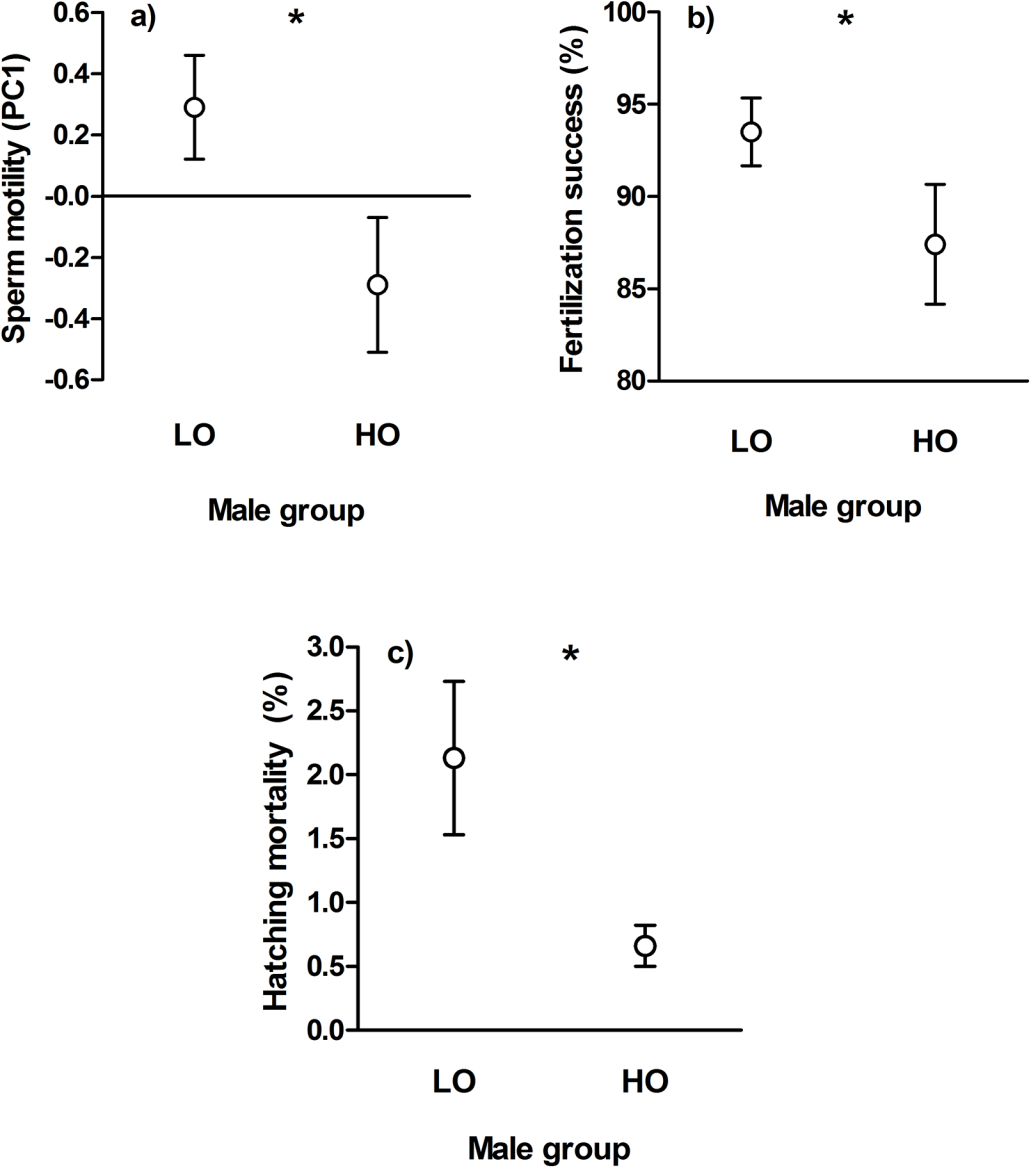
Sperm motility (a), fertilization success (b) and hatching mortality (c) differences (± S.E.) between less-ornamented (LO) and highly-ornamented (HO) males. Asterisks indicate statistically significant differences between male groups (*: *P* < 0.05).

Less-ornamented males had higher sperm motility than highly-ornamented individuals (LMM, PC1: d.f. = 23, t = -2,649, P = 0.014) (Fig 1A), but no statistically significant difference was found between groups in sperm swimming trajectory, although the curvature of sperm swimming pattern tend to be higher in highly-ornamented individuals (PC2: d.f. = 23, t = -1.734, P = 0.096). Less-ornamented males also had better fertilization success (LMM, d.f. = 23, t = -2.422, P = 0.024) (Fig 1B), but total egg mortality did not differ between male groups (LMM, d.f. = 119, t = 0.235, P = 0.82). Latter finding can be explained by the fact that egg hatching mortality was significantly higher among offspring of less-ornamented sires (GLMM, Z = -3.46, P < 0.001) (Fig 1C). Finally, larvae total length at hatching did not differ between male groups, although larvae tended to be larger among highly-ornamented sires (LMM, d.f. = 118, t = 1.704, P = 0.091).

### Association between sperm motility and offspring fitness

**Fig 2:**
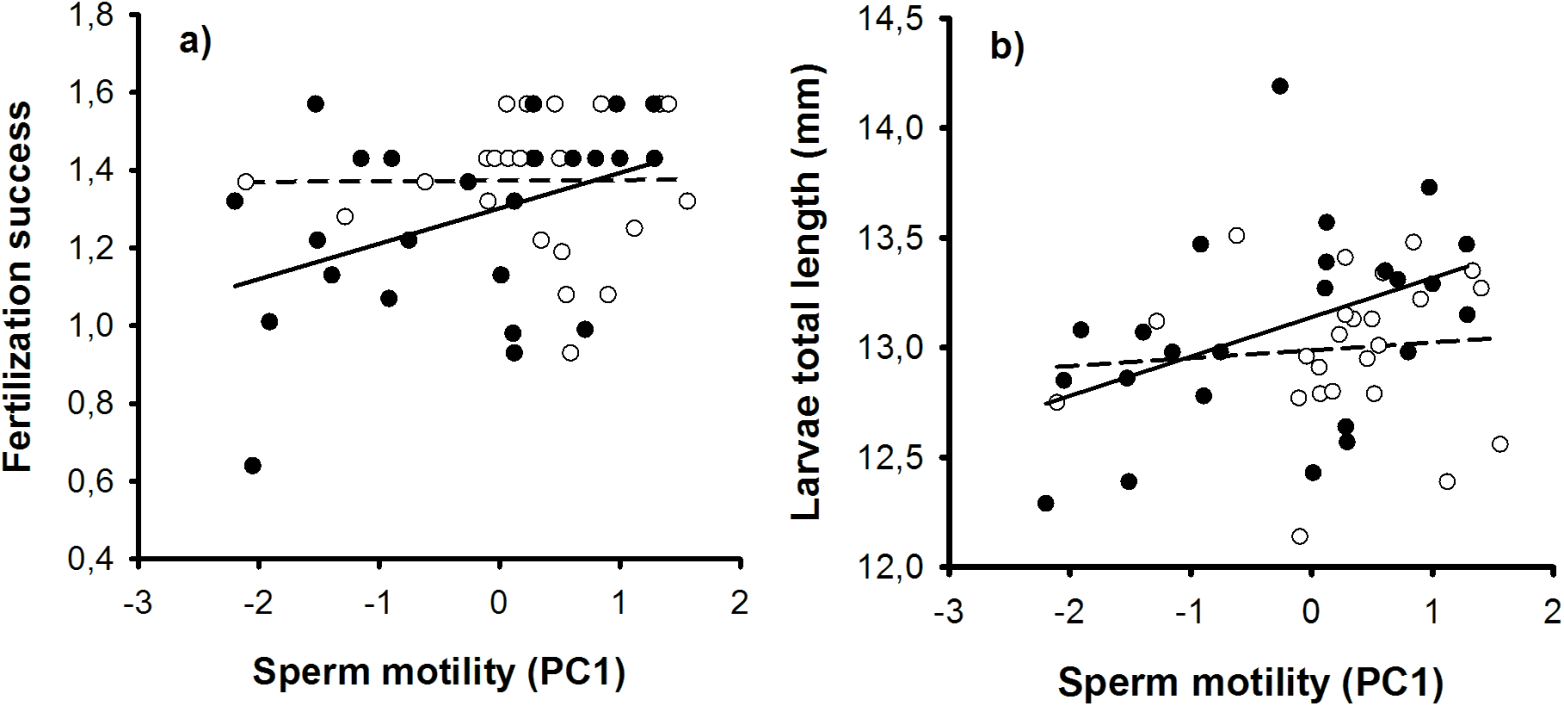
The association between sperm motility (PC1) and fertilization success (a) or larval total length (b). Less ornamented males = open dots + dashed line; highly-ornamented males = filled dots + solid line.

Sperm motility was not significantly associated with the fertilization success (LMM, d.f. = 42.49, t = 1.840, P = 0.073), egg hatching mortality or total egg mortality (removed from the final model). However, group-specific analyses revealed that sperm motility predicted sperm fertilization success in highly-ornamented males, i.e. the males with lower sperm motility (Pearson, r = 0.406, P = 0.049), but no association was found for less-ornamented males (r = 0.009, P = 0.967) (Fig 2A). Sperm motility was also positively associated with larvae total length (LMM, d.f. = 34.33, t = 2.263, P = 0.030) and the interaction between male groups and larvae total length was not significant (d.f. = 25.05, t = 1.803, P = 0.083). Group-specific analyses again showed that positive effect of sperm motility on larvae total length was statistically significant only in highly-ornamented males (Pearson, r = 0.439, P = 0.032), but not in less-ornamented individuals (r = 0.087, P = 0.687) (Fig 2B).

### Juvenile mortality, size and condition

**Fig 3:**
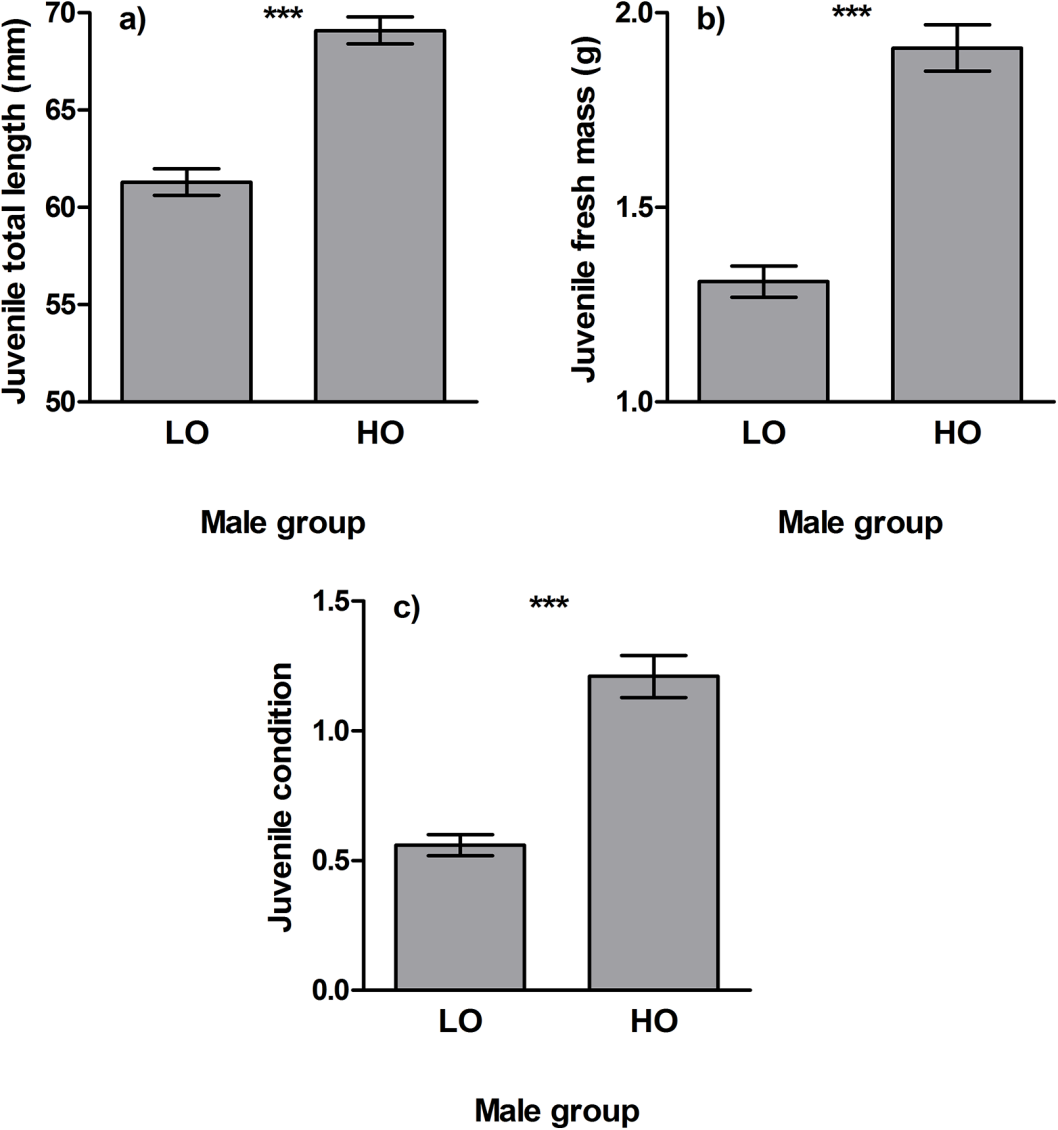
Juvenile total length (a), fresh mass (b) and condition (c) differences (± S.E.) between less-ornamented (LO) and highly-ornamented (HO) males. Asterisks indicate statistically significant differences between male groups (***: *P* < 0.001).

Juvenile (i.e. post-hatching) mortality of the offspring did not differ between highly-ornamented (mortality: 67.4%) and less-ornamented (mortality: 64.4%) males (Fisher’s Exact test, P = 0.389). However, offspring of highly-ornamented males were larger and had higher condition factor than offspring of less-ornamented individuals (One-Way ANOVA, total length: F_1,242_ = 63.8, P < 0.001; fresh mass: F_1,242_ = 70.2, P < 0.001; condition: F_1,242_ = 59.7, P < 0.001) (Fig 3).

## Discussion

In the present study males with less elaborate breeding ornamentation (subordinates) had higher sperm motility and also higher fertilization success than their highly-ornamented (dominant) conspecifics. On the other hand, offspring hatching success among less-ornamented males was significantly lower than in highly-ornamented males and consequently total offspring mortality did not differ between male groups. Furthermore, offspring size and body condition was found to be significantly lower in offspring of less-ornamented (subordinate) males, which may indirectly indicate that the social status may have a heritable basis. Since the mean total length and age (i.e. growth rate) of the males did not differ between the groups, we can rule out the possibility that observed differences between male groups and their offspring could be explained by male group-specific differences in mating tactics and thus age and size of the maturation (alternative mating tactics: see [37]).

Several studies have shown that males in disfavoured mating roles often have higher sperm swimming velocity than in males that have greater control over their paternities [7,10,15,17]. Accordingly, our finding that smaller (lower fresh mass) and less-ornamented (smaller breeding tubercles) males had higher sperm motility probably represents status-dependent difference in the investments in sperm motility [17,42]. We also found that subordinate males were more efficient in fertilizing the eggs than dominant males, but higher sperm motility did not result in higher paternity (hatching success of the larvae). This can be largely explained by the higher mortality during the hatching period (i.e. hatching mortality) among subordinate (less-ornamented) male offspring. The explicit selective mechanism behind this finding remains unknown. However, we observed an overall increase of water mould (*Saprolegnia* sp.) infection intensity in our incubating containers during the hatching period ([48], personal observations by the authors). This could indicate that the sperm of dominant (highly-ornamented) males produce offspring that are more resistant against *Saprolegnia*. Furthermore, we also found that sperm motility was positively associated with the egg fertilization success and larvae body size, but only in highly-ornamented males. Together these findings indicate that among subordinate males, selection may act in different directions at different life-history stages [49] and that sperm traits that have selective advantage at fertilization are not necessarily advantageous in later ontogeny [50].

Despite above-mentioned trade-offs, our results indicate that subordinate males may be able to (partly) compensate their disfavoured mating position by investing resources in highly motile sperm [51]. Furthermore, in the natural spawning situations subordinate males may also increase their fertilization success by investing more resources on sperm production (sperm quantity: [1]). Both of these factors may facilitate maintenance of less-ornamented male phenotypes in the wild, despite their reduced post-fertilization fitness. On the other hand, dominant males may be capable of compensating their less motile sperm by releasing their gametes closer and in better synchrony with the females [15]. Additional studies in fully natural breeding conditions would be required to further clarify the relative fitness of subordinate and dominant males in the wild.

We also found that the offspring of highly-ornamented males were larger and had better body condition than the offspring of less-ornamented males. In our earlier studies in whitefish we have found paternal effects both for larval size [44] and offspring early feeding success [43]. Furthermore, Huuskonen et al. [43] demonstrated that larvae first-feeding success is positively correlated with the size of the male breeding tubercles. Together these findings indicate that the offspring of highly-ornamented males are more efficient foragers than offspring of less-ornamented sires. Large body size may provide fish larvae a competitive advantage over smaller individuals [52,53] and is an important factor affecting larval survival in poor growth environments, that are common during natural (spring) hatching periods of whitefish larvae. Thus, in addition to the fact that dominant males may have better access to female gametes (see above), these males may be capable of compensate their impaired ability to fertilize the eggs also by producing offspring that have increased post-hatching fitness in the nature.

Together the present results suggest that male breeding tubercles may act as an honest signal of offspring fitness (growth rate, foraging success and possibly pathogen resistance). Furthermore, above-mentioned positive association between sperm motility and offspring fitness (egg fertilization success and larvae body size) in highly-ornamented males also partly support predictions of “good sperm” hypothesis [54,55,56]. In other words, among highly-ornamented males sperm phenotype was found to affect the phenotype of the resulting offspring, which suggest that sperm phenotype may be genetically correlated with offspring viability. However, our results also indicate that status-dependent differences in sperm motility may have important effects on offspring fitness in different life-history stages. Supporting this view, it has recently been demonstrated that a range of environmental effects can modulate sperm phenotypes and that such non-genetic variation can affect offspring phenotype and thus have important trans-generational consequences for offspring fitness [18,32,34,50,57,58]. Status-dependent plasticity in sperm traits seem to be common in salmonids and other externally fertilizing fish species [15,34,59]. Thus, it is possible that observed status-dependent sperm motility differences and resulting offspring fitness consequences may not be entirely genetically determined [34,35].

In conclusion, above-mentioned results suggest that male status and status-dependent differences in sperm traits may have important, but currently largely unknown consequences for offspring viability. Since the present study was based on split-clutch breeding design, where volume of sperm was equalized among all fertilizations, we can conclude that demonstrated effects most likely cannot be explained by female- or male-mediated material (gamete quantity) benefits. Further studies are needed to clarify the relative importance of genetic and environmental (non-genetic) effects behind our findings.

## Supporting Information

S1 Checklist The ARRIVE Guidelines Checklist. (PDF)
